# Management Challenges of Sleep-Disordered Breathing in a Child With Chronic Kidney Disease: A Case Report of a Child on Peritoneal Dialysis

**DOI:** 10.7759/cureus.60890

**Published:** 2024-05-23

**Authors:** Haneen M Toma, Amal R Al-Naimi, Ahmed Abushahin

**Affiliations:** 1 Pediatric Pulmonology, Sidra Medicine, Doha, QAT

**Keywords:** children, polysomnography, central sleep apnea, obstructive sleep apnea, sleep-disordered breathing, chronic kidney disease

## Abstract

Chronic kidney disease in children is a challenging condition that requires careful management. When combined with sleep-disordered breathing, it can pose even greater difficulties. This case report highlights the management challenges of a child with chronic kidney disease and sleep-disordered breathing. Through careful analysis and effective intervention, we were able to address the challenges and improve the child's quality of life. Understanding the complex interaction between these two conditions is crucial for healthcare professionals to provide effective care for children with chronic kidney disease and sleep-disordered breathing.

## Introduction

Sleep-disordered breathing (SDB) in children is a term used to describe sleep-related respiratory disorders that range from snoring to obstructive sleep apnea (OSA). SDBs are common in patients with chronic kidney disease (CKD). In adults, the reported prevalence of SDB ranges from 30% to 80% [1]. However, the prevalence of the condition in the pediatric population is uncertain and varies significantly, according to reports [2]. The relationship between CKD and SDB is complex. Having CKD can contribute to the development or worsening of sleep apnea. On the other hand, sleep apnea can potentially speed up the progression of CKD, ultimately increasing the likelihood of morbidity and mortality [3]. Managing sleep apnea in patients with CKD is extremely important to reduce the risk of related health problems and death. In this case study, we present the challenges associated with managing severe SDB in a pediatric patient suffering from CKD and on peritoneal dialysis.

## Case presentation

**Figure 1:**
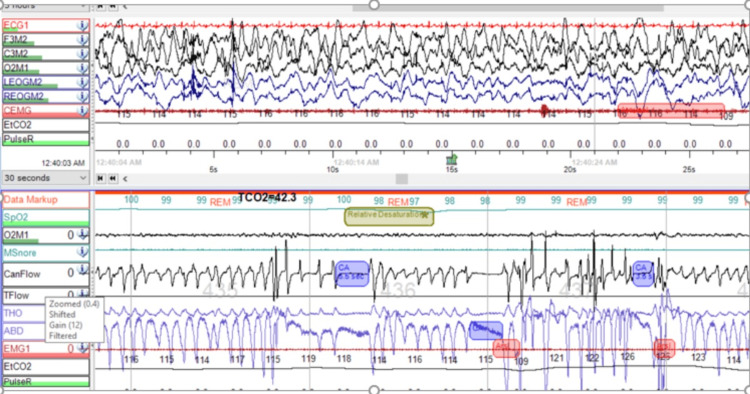
This diagnostic polysomnography for the patient was done off respiratory support and pre-intervention, which shows episodes of central apneas.

A nine-month-old full-term infant was born with multiple complex medical conditions, including posterior urethral valve, bilateral cystic dysplastic kidney, and pulmonary hypoplasia. Peritoneal dialysis was started on the fourth day of the baby's life. When the baby was five months old, the pulmonary service was consulted due to episodes of desaturation during sleep. However, the clinical examination did not reveal any signs of respiratory distress. The child's oxygen saturation level on room air was 98% daytime. No abnormalities in physical features, such as dysmorphic features or hypotonia, were observed. The child exhibited swelling throughout the body but had normal growth parameters. The weight was 8.4 kg at the 79th percentile, and the length was 65 cm at the 30th percentile. During the chest and cardiac examination, no abnormalities were found. The abdominal examination revealed ascites and bilateral inguinal hernia, but no signs of organomegaly. A polysomnography (PSG) was conducted to assess for SDB. The results of the PSG test showed an Apnea-Hypopnea Index (AHI) of 24 events per hour. The majority of the events were central hypopnea, with a central AHI (CAHI) of 24 events per hour. The condition was worse during rapid eye movement (REM) sleep, with a REM CAHI of 58 events per hour. The average oxygen saturation was 97%, with a nadir of 77%. Only 1.6% of the total sleep time was spent with oxygen saturation below 90%. The average end-tidal carbon dioxide (ETCO2) was 38 mmHg, with a peak of 59 mmHg. The ETCO2 level exceeded 50 mmHg for less than 1% of the total sleep time (Figure 1).

**Figure 2:**
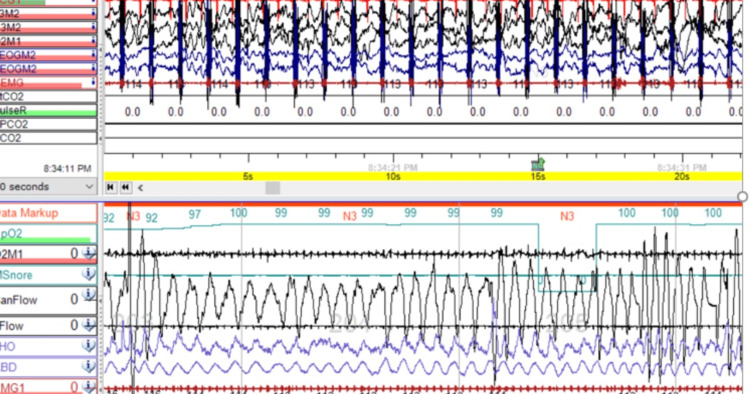
This diagnostic polysomnography for the patient was done off respiratory support and post-intervention, which shows normal sleep architecture with the resolutions of the sleep apnea events.

After examining the patient, several risk factors related to SDB were identified. The patient displayed a generalized swelling known as edema, which was affecting the head and neck areas, and was also suffering from severe gastroesophageal reflux disease (GERD). The patient had a large bilateral inguinal hernia, which was distended during peritoneal dialysis, making it difficult to position properly. The patient was undergoing nocturnal peritoneal dialysis, which can increase intra-abdominal pressure, limiting diaphragm excursion. Initial GERD treatment was unsuccessful, so a gastro-jejunal (G-J) tube was inserted for continuous overnight feeding with maximizing anti-reflux treatment. To avoid excessive fluid buildup in the body, the patient's peritoneal dialysis was switched to daytime, and a Dianeal solution is being used. Two weeks after making adjustments, a repeat PSG study showed a significant improvement in the patient's SDB. The AHI decreased to 1.9 events per hour and the REM AHI decreased to 6.3 events per hour. All respiratory events were central hypopneas. The average oxygen saturation was 99%, with a nadir of 91%. Additionally, all total sleep time was spent with oxygen saturation above 90%. The ETCO2 range was between 39.9 and 44.9 mmHg (Figure 2). 

As central hypopnea was predominant, a brain MRI was performed and revealed normal findings. After considering the severity of the SDB and the history of lung hypoplasia, an echocardiogram was performed which did not reveal any evidence of pulmonary hypertension.

## Discussion

CKD is characterized by structural or functional abnormalities in the kidneys resulting in a glomerular filtration rate (GFR) of less than 60 mL/min/1.73 m² for more than three months [4]. CKD patients are at risk for various sleep disorders including obstructive and central sleep apnea, hypoventilation, and restless leg syndrome. The prevalence of SDB in adults with CKD ranges from 30% to 80% [1]. In a recent meta-analysis, Kang et al. reported that the prevalence of SDB and OSA in pediatric patients was lower than expected. According to questionnaire studies, SDB and OSA had a prevalence of 21.7% and 34.0%, respectively. Furthermore, the study found that these sleep disorders were more common in dialysis-dependent patients compared to children who were not on dialysis [2]. Full-night PSG remains the gold standard for diagnosing SDB in children.

Amin et al. found that the Pediatric Sleep Questionnaire (PSQ) underestimated SDB in 19 children with stage 3-5 CKD, including seven patients who were dialysis dependent [5].

There are various theories to explain why SDB is common in people with CKD. One of the main factors is the presence of uremic toxins, which can damage the nervous system and muscles. This damage can cause fatigue in the respiratory muscles, including those that control the airway, leading to OSA [6]. Chemoreceptor responsiveness in CKD patients can be affected by various factors. Metabolic acidosis and uremia can lead to instability in ventilatory control. Metabolic acidosis, with or without compensated hypocapnia, can increase the chemoreceptor's sensitivity to carbon dioxide (CO2), causing changes in chemoreflex control. This process can eventually destabilize respiratory control during sleep by causing low levels of partial pressure of CO2, which can lead to CSA [7]. It is common for CKD patients to experience fluid overload, which can cause swelling of the pharyngeal muscles and lead to upper airway obstruction. This condition worsens when the patient is in the supine position. The accumulation of fluid in the lungs can stimulate pulmonary mechanoreceptors, which can trigger hyperventilation and ultimately lead to central sleep apnea (CSA) [8]. Patients with chronic kidney disease may experience nocturnal hypoventilation due to the bicarbonate-based dialysis solution, resulting in metabolic alkalosis [9].

Studies have indicated a reciprocal relationship between OSA and CKD, with a higher prevalence of CKD among adult patients with OSA [3]. This association could be due to the hypoxia associated with SDB. OSA, in particular, disrupts the balance between reactive oxygen species production and elimination, which can lead to structural and functional changes in the kidney [10]. Children affected by OSA are at risk of developing systemic hypertension. SDB can cause hypercapnia and hypoxia, which in turn increase sympathetic tone. This activates the renin-angiotensin-aldosterone system (RAAS) and causes endothelial dysfunction and oxidative stress. Ultimately, these processes lead to cardiovascular consequences, including hypertension [11].

Effective management of SDB in patients with CKD entails a thorough assessment of remediable causes, which encompasses evaluating factors such as obesity and anatomic abnormalities in the upper airway, such as adenotonsillar hypertrophy. It is important to carefully choose the type of dialysis fluids since bicarbonate-based fluids can lead to hypoventilation [8]. Patients with moderate to severe SDB should consider continuous positive airway pressure (CPAP) therapy to reduce complications. Koga et al. conducted a study on adult patients with OSA and CKD and found a significant improvement in renal plasma flow and filtration fraction after initiating CPAP therapy [12].

Renal transplantation is a medical procedure that partially restores kidney function and helps to improve many uremic symptoms and complications. However, there is limited data available on SDB in renal transplant patients. Existing studies have relied on sleep quality questionnaires, which generally show improved sleep quality in adult transplanted patients [13]. Ogna et al. reported a statistically significant decrease in AHI six months after renal transplant in 42 adult patients with CKD based on home nocturnal PSG [14].

We took a comprehensive approach to address the factors contributing to the patient's SDB. An evaluation by ENT specialists ruled out adenotonsillar hypertrophy. Due to the severity of GERD, the patient underwent G-J tube insertion. The dialysis fluid used was dextrose-based, and the patient's peritoneal dialysis timing was changed from nocturnal to daytime. After these interventions, subsequent repeated PSG showed complete resolution of SDB, and the patient did not require nocturnal non-invasive ventilation (NIV) support.

## Conclusions

In conclusion, diagnosing SDB in pediatric patients with CKD is a challenging task. The symptoms of SDB and CKD overlap, such as daytime fatigue, sleepiness, and cognitive impairment. Therefore, a careful evaluation of medical history and a thorough examination, along with a full-night PSG in suspected cases, is recommended. Managing SDB in CKD patients requires a comprehensive assessment of all possible factors contributing to SDB before initiating NIV support. Further, PSG-based studies are necessary to report the prevalence of SDB in pediatric CKD patients and to determine the effect of CKD management on SDB prevalence.
